# Affinity biosensors using recombinant native membrane proteins displayed on exosomes: application to botulinum neurotoxin B receptor

**DOI:** 10.1038/s41598-017-01198-1

**Published:** 2017-04-21

**Authors:** Richard Desplantes, Christian Lévêque, Benjamin Muller, Manuela Lotierzo, Géraldine Ferracci, Michel Popoff, Michael Seagar, Robert Mamoun, Oussama El Far

**Affiliations:** 1 INSERM, UMR_S 1072, 13015 Marseille, France; 2 Aix-Marseille Université, 13015 Marseille, France; 3 Ciloa, cc90 - Université Montpellier 2, Place E. Bataillon, 34095 Montpellier Cedex 5, France; 4 CNRS, UMR 7286, Plate-Forme de Recherche en Neurosciences PFRN, 13015 Marseille, France; 5 CNR Anaérobies et botulisme, Unité Bactéries anaérobies et toxines. Institut Pasteur, 28 rue du Dr Roux, 75724 Paris, Cedex 15 France

## Abstract

The development of simple molecular assays with membrane protein receptors in a native conformation still represents a challenging task. Exosomes are extracellular vesicles which, due to their stability and small size, are suited for analysis in various assay formats. Here, we describe a novel approach to sort recombinant fully native and functional membrane proteins to exosomes using a targeting peptide. Specific binding of high affinity ligands to the potassium channel Kv1.2, the G-protein coupled receptor CXCR4, and the botulinum neurotoxin type B (BoNT/B) receptor, indicated their correct assembly and outside out orientation in exosomes. We then developed, using a label-free optical biosensor, a new method to determine the kinetic constants of BoNT/B holotoxin binding to its receptor synaptotagmin2/GT1b ganglioside (k_on_ = 2.3 ×10^5^ M^−1^.s^−1^, k_off_ = 1.3 10^−4^ s^−1^), yielding an affinity constant (K_D_ = 0.6 nM) similar to values determined from native tissue. In addition, the recombinant binding domain of BoNT/B, a potential vector for neuronal delivery, bound quasi-irreversibly to synaptotagmin 2/GT1b exosomes. Engineered exosomes provide thus a novel means to study membrane proteins for biotechnology and clinical applications.

## Introduction

Determination of the binding kinetics of analytes is potentially extremely useful for drug development and assay validation. In principle, purified endogenous or recombinant membrane proteins can be integrated into a lipid environment, such as proteoliposomes, supported lipid bilayers or nanodiscs. However, this approach is not ideal, due to the fact that membrane proteins are difficult to produce, prone to denaturation and do not always retain their functional integrity in artificial lipid bilayers^1, 2^.

Exosomes are extracellular vesicles, produced by many cell types and can be detected or purified using antibodies^3, 4^. Furthermore, the ability of exosomes to carry molecules from native or engineered parental cells and fuse with recipient cells to deliver their cargo, confers considerable biomedical potential^5, 6^. Unlike intracellular vesicles, exosomes display homogeneous topology in which plasma membrane proteins have the same outside-out orientation as at the surface of intact cells. Moreover, exosomes can potentially provide the means of expressing recombinant proteins in small membrane vesicles that can be used as vaccine strategies^7, 8^.

Our principal aim was to develop a general approach to address recombinant membrane proteins to exosomes, using a specific targeting sequence to direct exosomal sorting of a set of membrane drug targets, with a particular focus on the botulinum neurotoxin/B (BoNT/B) receptor. BoNT serotypes A, B and E (BoNT/A, BoNT/B, BoNT/E), produced by the bacteria *Clostridium botulinum* are the main cause of human botulism and are produced as a complex of holotoxin (MW 150 000 Da) and several nontoxic proteins. Although BoNTs are among the most neurotoxic substances known, their ability to reversibly block cholinergic nerves has provided the basis for treatment of human diseases^9^. Therapeutic potential is currently being extended by the discovery of subtle differences in BoNT subtypes and the development of engineered BoNTs^10^.

At the molecular level, BoNTs are di-chain proteins composed of a heavy chain (Hc, 100 kDa) and a light chain (Lc, 50 kDa), linked by a disulfide bridge and non-covalent interactions^11^. The Hc mediates binding at the presynaptic surface and internalization by receptor-mediated endocytosis, followed by translocation of the light chain through the synaptic vesicle membrane into the cytoplasm. The Lc is a zinc-dependent endopeptidase which cleaves host proteins essential for neurotransmitter release, resulting in muscle paralysis^9^. The Hc domains of BoNT/A, B and E contain binding sites for a polysialo-ganglioside, in particular GT1b and a protein receptor, which confer neuro-specific binding with K_D_s in the 0.5 nM range^12–14^. The protein receptors for BoNTs are intra-luminal domains of transmembrane synaptic vesicle proteins, which become accessible to the extracellular milieu after synaptic vesicle fusion. BoNT/A and BoNT/E share SV2 protein as their receptor, whereas BoNT/B binds to synaptotagmin 1 or 2 (SYT1 or 2), but has a higher affinity for SYT2^11, 15^. The standard method for quality control of pharmaceutical batches of BoNT is the mouse toxicity bioassay, which requires a large number of animals and is ethically controversial. Surrogate molecular assays capable of measuring the functionality of BoNT Hc and Lc *in vitro* are therefore urgently required. Highly sensitive assays for the enzymatic activity of the Lc from BoNT/A, B and E have been established^16–18^. However, methods allowing detailed analysis of the binding parameters that determine neuro-specificity are lacking.

This study describes a new method to express full-length complex membrane receptor proteins targeted to exosomes. The exosomes were validated using conformation-dependent ligand binding to the voltage-gated potassium channel Kv1.2 and the G-protein coupled receptor CXCR4 and used to determine the kinetic and equilibrium binding constants of BoNT/B to its receptor.

## Results

### Exosomal expression of membrane proteins

**Figure 1:**
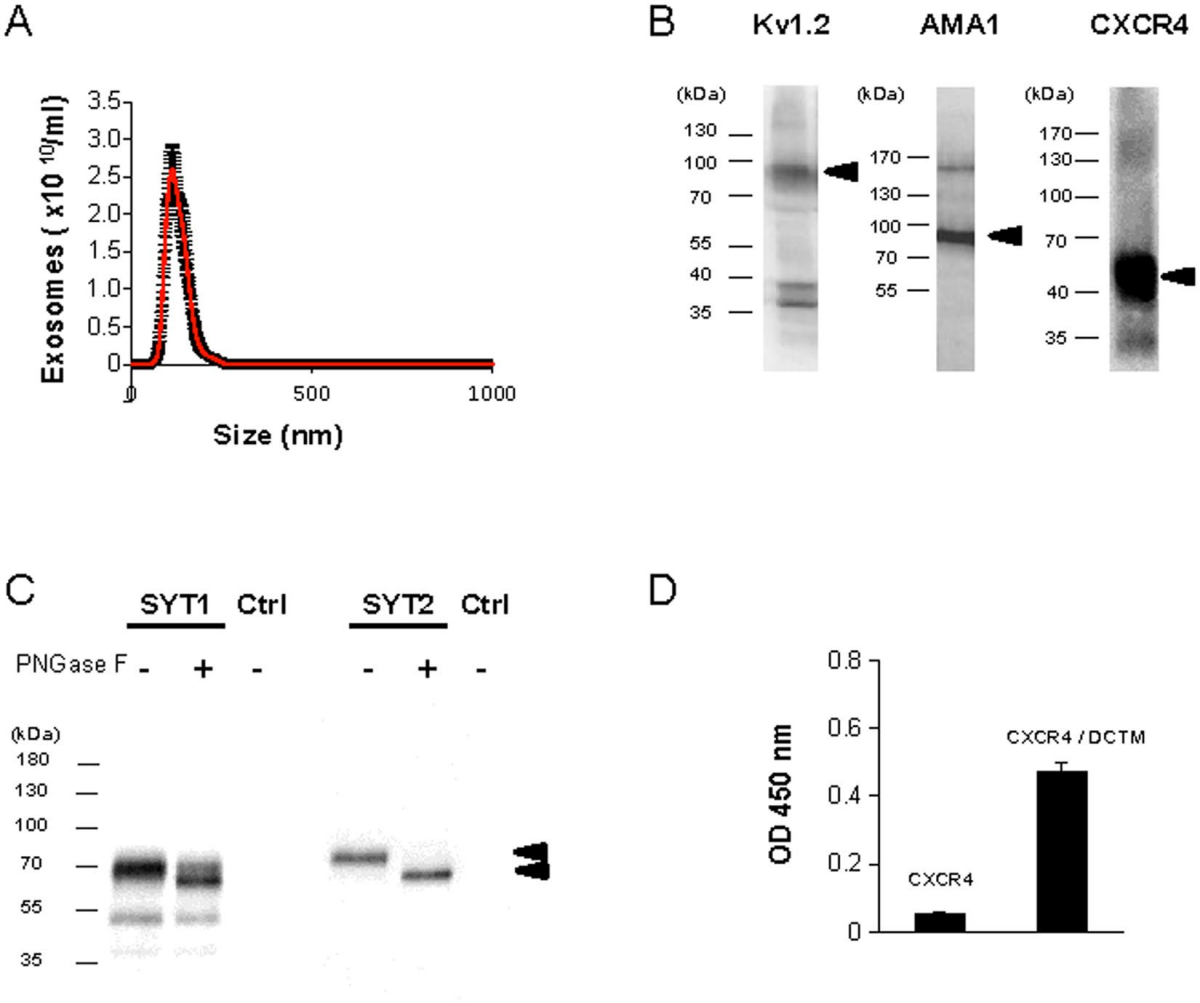
Expression of recombinant membrane proteins in exosomes. (**A**) Nanoparticle tracking analysis of purified exosomes yielded a mean size of SYT2-expressing exosomes of 127 ± 30 nm (mean of 5 independent determinations). (**B**) Kv1.2, AMA1 and CXCR4 exosomal preparations were analyzed by Western blot using specific antibodies. Arrowheads indicate expected molecular weight. (**C**) ExoSYT1 and 2 were treated in the presence or absence of PNGase F and analyzed by Western blot using anti-SYT antibody (1D12). Exocontrol from non-transfected cells were used as a control (Ctrl). (**D**) Expression levels of CXCR4 in exosomal preparations with or without the presence of the DCTM peptide tag. The nonspecific signal has been subtracted. Error bars are SD from triplicates. Representative of 3 experiments using 2 independent exosomes preparations.

A patented technology which sorts chosen membrane protein to exosomes, was used to express 5 different transmembrane proteins: the BoNT/B receptors synaptotagmin 1 and 2, the seven transmembrane domain G-protein-coupled receptor CXCR4, the voltage-gated potassium channel Kv1.2, and the type 1 membrane protein *Plasmodium falciparum* apical membrane antigen (AMA1). A sequence encoding a peptide (DCTM), which promotes exosomal sorting^19^ (patent no. WO2009115561), was fused to the 3′ end of each coding sequence cDNA and the resulting plasmids transfected in HEK cells. We established stable cell lines for synaptotagmins (SYTs) and transient expression for the other membrane proteins. Extracellular vesicles (exoKv1.2; exoCXCR4; exoAMA1; exoSYTs) were recovered from transfected HEK cell culture media and purified. The size of the membrane particles was evaluated by nanoparticle tracking analysis, yielding a mean diameter of 127 nm (Fig. 1A) for exoSYT2, consistent with the size of exosomes from HEK cells^20^.

Exogenously expressed proteins were detected in purified exosomal fractions by Western blotting (Fig. 1). All observed molecular weights were compatible with the predicted size of the mature full-length recombinant proteins (arrowheads, Fig. 1B,C). PNGase F treatment of exoSYT1/2 resulted in an increase in mobility, indicating N-glycosylation (Fig. 1C).

It has been previously shown that structurally complex proteins, such as G protein-coupled receptors, are addressed to exosomes when transiently transfected in cultured cells (Estelle 2005). We thus evaluated the relevance of our approach in the case of CXCR4 and compared its expression level, in the presence or absence of the DCTM tag. Exosomes purified from CXCR4 or CXCR4-DCTM transfected cells were coated in ELISA plates and CXCR4 expression was measured using anti-CXCR4 antibody. As shown in Fig. 1D, addition of the DCTM tag resulted in an approximately 10-fold (9 ± 0.8, n = 3 from 2 independent exosome preparations) increase in the expression of CXCR4.

### Immunocharacterization of recombinant exosomes

**Figure 2:**
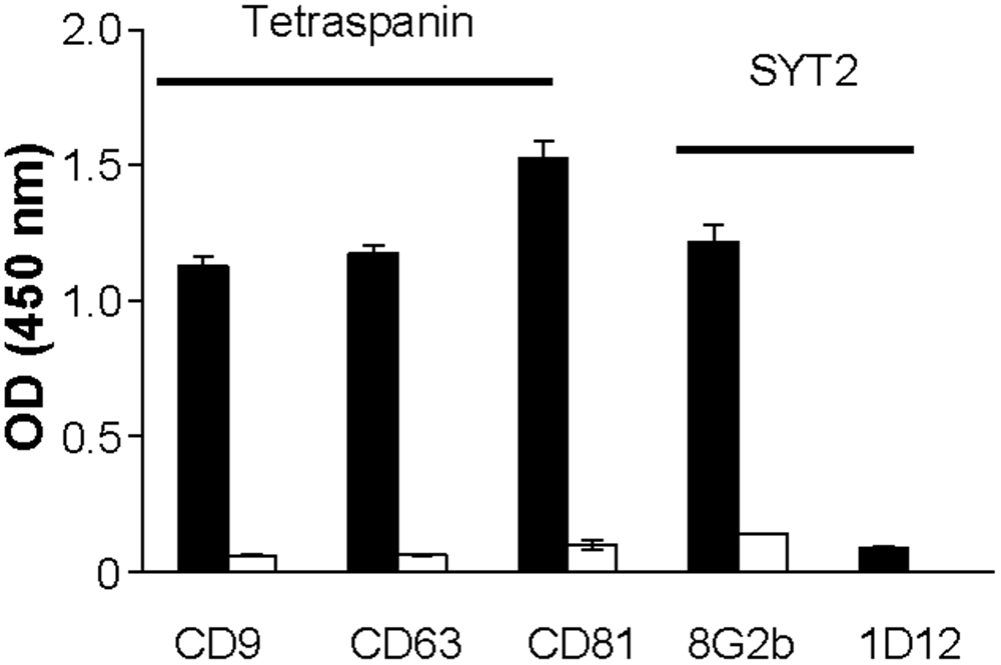
Characterization of SYT2-expressing exosomes. ExoSYT2 were captured on ELISA plates coated with anti-SYT2 (black bars) or anti-GST negative control antibodies (open bars). The presence of exosomal markers of the tetraspanin family (CD9, CD63 and CD81) was assessed using the corresponding antibodies. The orientation of SYT2 on captured exosomes (using mAb8G2b and mAb1D12 recognizing respectively the N-terminal and the cytosolic domains of SYT2) was verified. Error bars are SD from triplicates. Representative of 3 independent experiments.

To confirm the identity of the secreted membrane vesicles, we investigated the co-expression of exosomal surface markers in exoSYT2. Secreted membrane particles were captured on ELISA plates using specific antibodies and immobilized particles were probed with mAbs directed against exosomal surface markers. As shown in Fig. 2, immunocaptured SYT2 exosomes expressed three classical exosomal markers CD9, CD81 and CD63^21^. Similar results were obtained using exoCXCR4 (data not shown). A mAb directed against the N-terminal domain of SYT2, which is intraluminal in synaptic vesicles and extracellular at the neuronal plasma membrane recognized the immobilized particles in contrast to an absence of recognition by mAb1D12 that recognizes its cytoplasmic domain. These data indicated that as expected, the transmembrane orientation of SYT2 was identical to that in neuronal plasma membranes.

Surface plasmon resonance (SPR) was used to evaluate the expression level of recombinant SYT2 or CXCR4 in immunocaptured exosomes. Exosomes were specifically and stably captured on sensorchips and non-specific capture on control IgG was less than 10% of the total signal (Supplementary Fig. 1). Anti-SYT2 antibody and a conformation-dependent anti-CXCR4 antibody were then injected at saturating concentrations. mAb binding responses (Rmax) reflected thus the total quantity of immobilized receptor. We found 0.07  RU (resonane units) of specific mAb/RU of immobilized exosomes for exoSYT2 and 0.1RU/RU for exoCXCR4, yielding a mean of 0.08 +/− 0.01 RU (n = 4) of bound antibody per RU of immobilized exosomes (not shown). Assuming that 1RU of mAb bound = 1 pg/mm^2^ of molecule^22^ on the exosome surface, a protein to lipid ratio of 2:1 (w:w) and that the molecular weight of SYT2/DCTM is 53 000 Da, one can calculate a content of 0.7–1.4 nmoles of SYT2/mg of protein. SYT2 would thus represent 3.6–7.2% of total proteins in SYT2 exosomes, depending on whether the mAb/SYT2 interaction is monovalent or bivalent. A comparison of this value with our previous results obtained in similar conditions with synaptic vesicles^22^ suggests that each exosome contains about 200 copies of exogenously expressed protein (see supplementary Methods).

### Ligand binding to membrane proteins expressed in exosomes

**Figure 3:**
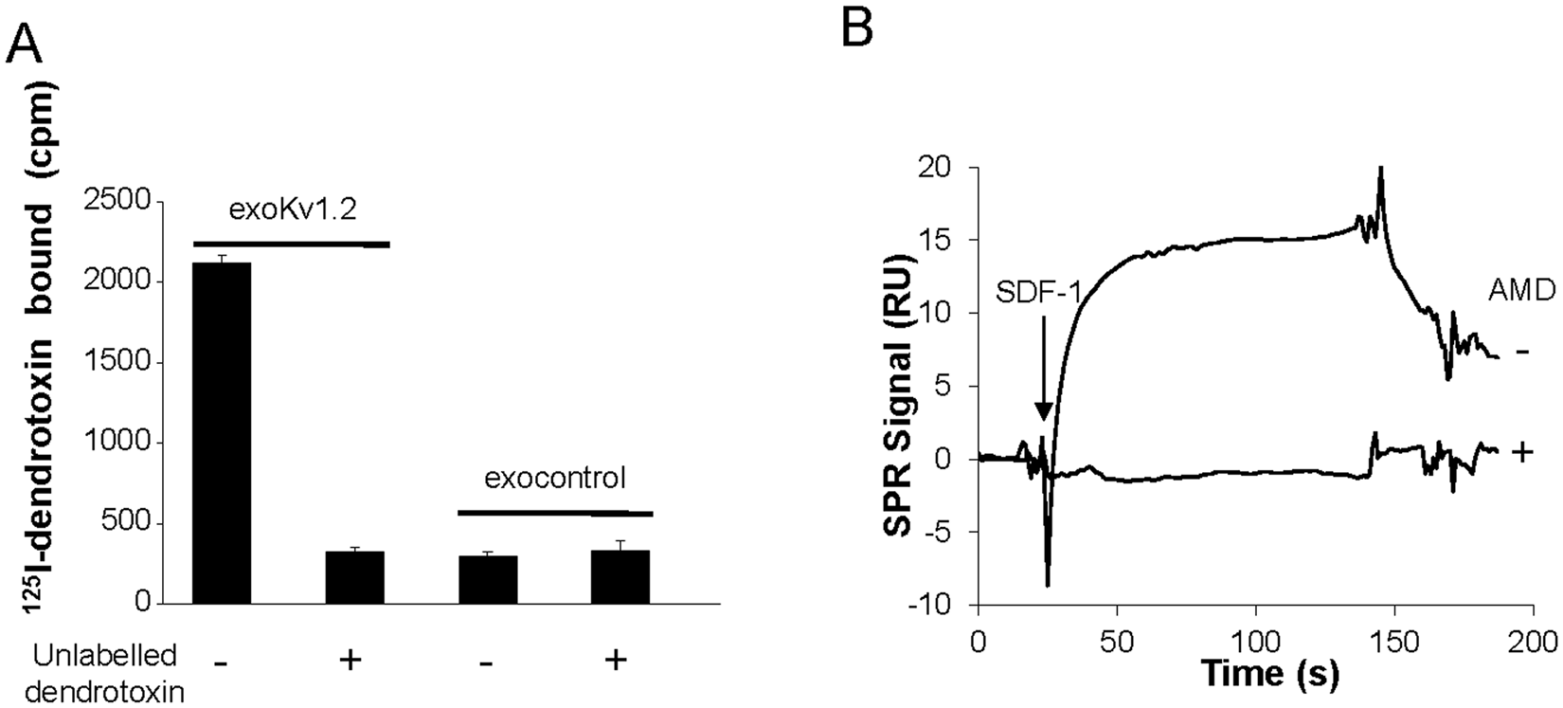
Measurement of ligand binding to exoKv1.2 and exoCXCR4. (**A**) Binding of ^125^I-dendrotoxin to exoKv1.2. Exosomes were incubated with ^125^I-dendrotoxin in the absence or presence of an excess of unlabelled dendrotoxin and bound toxin counted (cpm = counts per minute). Error bars are SD from triplicates. Representative from 4 independent experiments. (**B**) SDF1 binding to CXCR4 exosomes. The CXCR4 ligand SDF-1 (50 nM) was injected over exoCXCR4 immobilized on a chip. SDF-1 binding was measured and its specificity confirmed using the CXCR4 antagonist AMD3100 (10 µM). Note that, as SPR monitors the mass of bound analyte, AMD3100 binding was not detected owing to it’s low molecular mass relative to SDF-1. Representative SPR sensorgrams, after blank subtraction, from 3 independent experiments.

We used the binding of specific ligands to address the proper assembly and/or conformation of the tetrameric Kv1.2 channel or CXCR4 at the surface of exosomal membranes. Radiolabeled ^125^I-dendrotoxin, a specific ligand that binds to an extracellular site on the Kv1.2 channel, was incubated with exoKv1.2, in the presence or absence of an excess of unlabelled toxin. Exosomes were then filtered to separate free and bound toxin. While dendrotoxin did not bind to exocontrol, robust specific binding to exoKv1.2 was measured (Fig. 3A). Excess unlabelled dendrotoxin competitively inhibited binding, reaching the background signal determined using control exosomes (Fig. 3A).

SPR spectroscopy was used as a label-free sensing method to measure the conformationally-sensitive binding of SDF-1 to exoCXCR4^23^. To measure SDF-1 binding, sensor chips were functionalized independently on separate flowcells, with exoCXCR4 or exocontrol. SDF-1 binding to exoCXCR4 was quantified by automatically subtracting the non-specific signal obtained with exocontrol. As shown in Fig. 3B, SDF-1 (8 kDa) specifically bound to exoCXCR4 but binding was abolished if SDF-1 was injected in presence of the competitive antagonist AMD3100 (794 Da). These specific ligand-binding experiments suggest that recombinant proteins are expressed at the surface of exosomes with appropriate membrane topology and conformation.

### ELISA detection of BoNT/B binding to exo SYT1/2

**Figure 4:**
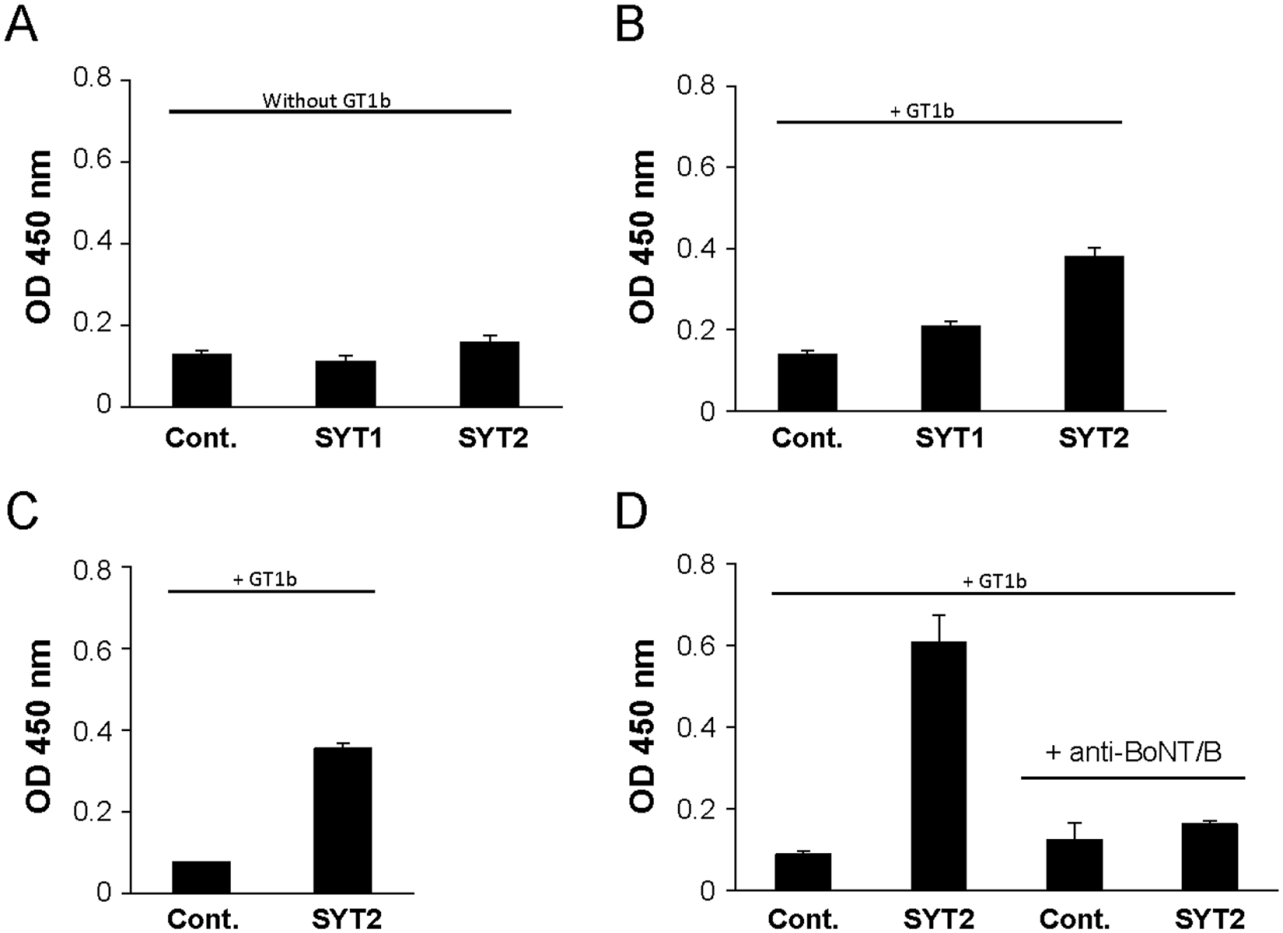
ELISA detection of BoNT/B interaction with exoSYT. (**A**,**B**) ExoSYT1, exoSYT2 and exoAMA1 (Cont) were directly coated on ELISA plates then loaded with (**B**) or without (**A**) GT1b and incubated with BoNT/B (10 nM). Binding was revealed using an anti-BoNT/B antibody. Results are representative of 5 (p > 0.05) (**A**) and 6 (p < 0.05) (**B**) independent experiments. (**C**) HcB (10 nM) binding to exoSYT2/GT1b was measured as described for BoNT/B representative of 2 independent experiments. (**D**) Same as in (**B**) but binding was performed in presence of 25% human serum. BoNT/B binding specificity was assessed using BoNT/B pre-incubated with affinity-purified anti-BoNT/B neutralizing antibodies. Error bars are SD from triplicates. Representative of 3 independent experiments using different sera.

ELISA experiments showed that both GT1b and GD1a, the two main gangliosides that cooperate with SYT to form the high affinity BoNT/B binding site, were absent in exosomal preparations (Supplementary Fig. 2). Accordingly, no specific BoNT/B binding was measured by ELISA upon direct coating of exoSYT1, exoSYT2 when compared to the negative control exoAMA1 (Fig. 4A, p > 0.05, n = 5 independent experiments). As GT1b can spontaneously insert in lipid membranes, exosomes were pre-incubated with GT1b before BoNT/B binding. Under these conditions, BoNT/B binding was detected at the surface of exoSYT1/GT1b and exoSYT2/GT1b (p < 0.05, 6 independent experiments, Wilcoxon matched pairs test). In accordance with the reported lower affinity of BoNT/B for SYT1 versus SYT2, BoNT/B binding to exoSYT1/GT1b was weaker, compared to exoSYT2/GT1b (Fig. 4B). The recombinant form of the C-terminal domain of BoNT/B (HcB) also bound to exoSYT2/GT1b (Fig. 4C). In contrast, BoNT/A did not bind neither to exoSYT2 nor to exoSYT1 in presence or absence of GT1b (Supplementary Fig. 3). We checked if the ELISA detection of BoNT/B interaction with its receptor could be achieved in the presence of serum as the enzymatic activity of BoNT/B *in vitro* is largely inhibited under these conditions^16^. BoNT/B binding to SYT2/GT1b exosomes was readily revealed in the presence of 25% serum (Fig. 4D) and abrogated by pre-incubation of the toxin with neutralizing antibodies^16^, confirming binding specificity. Binding with serum gave higher signals than in its absence suggesting that unknown factors present in sera potentiate binding.

### Biosensor determination of BoNT/B binding parameters in SYT2 exosomes

**Figure 5:**
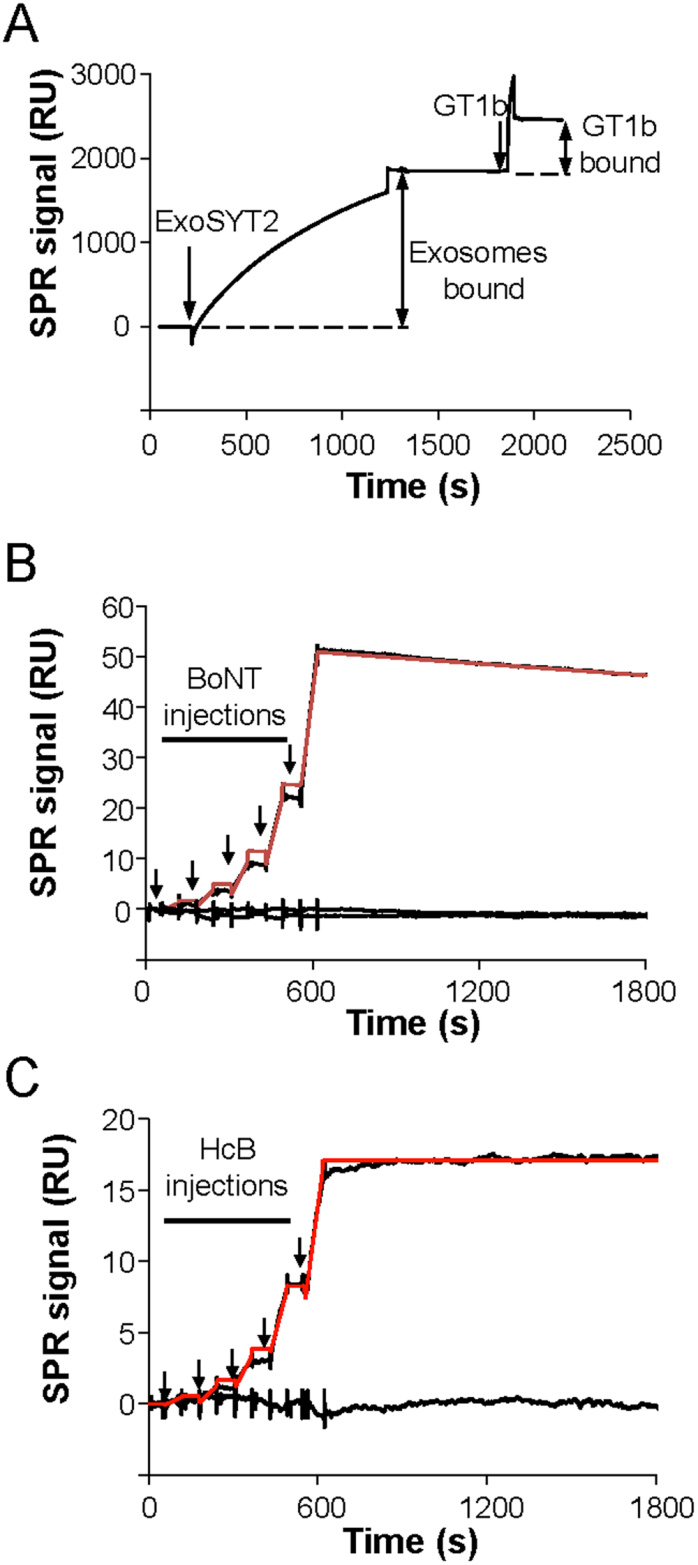
SPR measurements of BoNT/B and HcB affinity on immobilized exoSYT2/GT1b. (**A**) Functionalization of exoSYT2 flowcell: ExoSYT2 were injected into the flow-cell of a sensor chip with coupled anti-SYT mAb. Subsequent injection of GT1b (1 µM) yielded an increase in RU illustrating online ganglioside insertion in exosomal membranes. (**B**) BoNT/B affinity determination: SPR analysis of the binding of BoNT/B holotoxin injected (arrows) in 2-fold dilutions from 1 to 16 nM to immobilized exoSYT2/GT1b and exoAMA1/GT1b. Data obtained on exoAMA1 were automatically subtracted from exoSYT2 measurements and only specific data presented. The red trace corresponds to fitted data. The lower traces correspond to injection of BoNT/A and BoNT/E holotoxins to exoSYT2. (**C**) HcB affinity determination: As in (**B**) using HcB (arrows) instead of BoNT/B. Red trace corresponds to theoretical fitting. The lower trace corresponds to specific measurement of HcA binding to exoSYT2.

The binding affinity of BoNT/B was analyzed by SPR. ExoSYT2 and exoAMA1 were captured on sensor chips in separate flow cells, and GT1b was briefly loaded on immobilized exosomes before BoNT/B injection (Fig. 5A). Kinetic experiments were carried out by delivering increasing concentrations of BoNT/B, in a continuous flow, over both exoSYT2 and control exosomes. As BoNT/B binds the immobilized partner, the mass of material bound specifically to the surface increases with concentration as illustrated in Fig. 5B. Responses fit well to a 1:1 interaction model yielding a K_D_ = 0.6 ± 0.1 nM (mean ± sem from 6 independent experiments) for BoNT/B holotoxin with an association rate constant (k_on_) of 2.3 ± 1.10^5^ M^−1^ s^−1^ and a dissociation rate constant (k_off_) of 1.3 ± 0.5 10^−4^ s^−1^ (Table 1). Similar values were obtained for BoNT/B (Table 1). Interestingly, while BoNT/B dissociated slowly (Fig. 5B), the isolated binding domain of BoNT/B (HcB) showed different kinetic characteristics (Fig. 5C). HcB bound very tightly to exoSYT2/GT1b with a k_off_ < 10^−5^ s^−1^, precluding a precise affinity determination, as k_off_ was under the limit of detection of the apparatus. Flat sensorgrams obtained upon injection of increasing concentrations of BoNT/A, BoNT/E holotoxins or HcA over exoSYT2, illustrate binding specificity (Fig. 5B and C).

**Table 1:** Affinity constants of BoNT/B and HcB for exoSYT2/GT1b.

Toxin (subtype)	M W (kDa)	Activity (LD_50_/mg)	k_on_ × 10^5^ (M^−1^.s^−1^)	k_off_ × 10^−4^ (s^−1^)	K_D_ (nM)	n
BoNT/B holotoxin (B1)	150	0.5 × 10^8^	2.3 ± 1	1.3 ± 0.5	0.6 ± 0.1	6
BoNT/B (B1)	550	1.2 × 10^7^	3.1 ± 1.2	4.1 ± 1.4	1.4 ± 0.2	4
HcB (B2)	50	—	nd	<0.1	nd	4

n = independent experiments; nd = not determined; Values are mean ± SEM.

The equilibrium dissociation constant obtained from the kinetic parameters of BoNT/B binding to exoSYT2/GT1b was very close to reported K_D_s measured at equilibrium using native nerve terminals^12, 14^, thus validating the exosome biosensor approach. However it should be noted that binding assays require nanomolar toxin concentrations and are not adapted to the detection of extremely low BoNT/B concentrations for diagnostics and surveillance.

## Discussion

There is an increasing demand for simple molecular assays to measure precise binding properties of ligands to membrane receptors that are major drug targets. However, successful reconstitution of functional membrane proteins is an empirical process, depending on many factors that must be optimized, including the formation and composition of the lipid environment^2^. We encountered this problem while developing cell-free biosensor assays to measure the affinity and kinetics of BoNT interactions with their membrane receptors. Reported binding assays, using full-length proteins or soluble domains of BoNT receptors, yielded affinities considerably lower than those found at synaptic terminals^24–27^. In addition, cellular models are cumbersome and less suitable for reproducible affinity measurements. Exosomes potentially provide an ideal mini-cell-like model, due to their stability, with a high cholesterol content, and a uniform native membrane receptor orientation, with post-translational modifications. Several reports indicated that exosomes can be modified to express soluble proteins^6, 7^, however data concerning membrane protein targeting to exosomes are lacking. De Gassart and al.^19^, have demonstrated that the cytosolic domain of the transmembrane envelope (Env) protein of bovine leukemia virus contains motifs (DCTM) that potentially interact with the Endosomal Sorting Complexes Required for Transport (ESCRT) machinery, and can be used to address the ecto-domain of CD8 to exosomes. We therefore set out to develop a novel general approach to address membrane proteins to exosomes for simple assays, which can be automated and applied, in many laboratory settings. We confirm and extend the use of this exosomal targeting sequence (DCTM) to express the voltage-gated potassium channel Kv1.2, the G-protein coupled receptor CXCR4, as well as the BoNT/B receptors SYT1 and SYT2. All recombinant proteins were properly oriented in vesicles which expressed typical tetraspanin exosomal markers. However, we did not investigate whether the transfected proteins were expressed in all exosomes or whether they were also present in other types of secreted vesicular particles. SPR chips were functionalized with specific antibodies, allowing exosomes to be captured and stably immobilized. As little as 500 pg of immobilized exosomes were enough to measure BoNT/B binding. We estimate that there are about 200 copies of SYT2 or CXCR4 per exosome. This high copy number was indirectly confirmed by SPR measurement of binding of the relatively small analyte SDF-1 (8 kDa) to CXCR4 exosomes. Our method clearly increased specific targeting to exosomes, compared to viral overexpression of a GPCR in the absence of a particular tag^28^.

Expression of the homo-tetrameric Kv1.2, CXCR4 and botulinum neurotoxin type B (BoNT/B) receptors in functional conformation was demonstrated in several binding assay formats: filtration assay, ELISA and SPR. The potential of this methodology was illustrated by measuring BoNT/B binding by ELISA and SPR to a double receptor consisting of SYT and the ganglioside GT1b. The ELISA method is effective in the presence of serum and could therefore be used to develop anti-BoNT/B antibodies, which are currently the only treatment for intoxication^29^ and to screen for new antidotes acting on either the native receptor or the toxin. The exosome-based method, with a fully native double receptor, represents a flexible and more physiological context than minimal BoNT binding peptides. Furthermore, repeated injections of BoNT/B for clinical applications produce anti-toxin antibodies in a small number of patients^30^. The exosome assay could be used to determine whether these antibodies reduce therapeutic efficacy by perturbing BoNT/B binding to its receptor. It is however important to note that the exosome-based assay is not sensitive enough for direct detection of BoNT/B in the serum of patients with type B botulism, as toxin concentrations in this case are very low. Multi-step, sensitive (fM to pM) methods are commonly used to reveal, through the detection of its enzymatic activity, BoNT/B binding to native receptors^31^ or recombinant peptide-based receptors^32, 33^. A consecutive enzymatic assay should significantly increase the sensitivity of our system^16, 34^. The native double receptor for BoNT/B i.e. synaptotagmin/GT1b was successfully reconstituted yielding K_D_s similar to those measured using native membranes. Studies using SYT peptides have always yielded lower affinities^24, 26, 27^ and fail to reconstitute the double receptor. However, due to the native origin of exosomes, the copy number of BoNT receptors per exosome is significantly lower than peptide concentrations that can be coated on beads. Therefore, this test is neither appropriate for the detection of minute amounts of BoNT nor can it recapitulate all the BoNT intoxication steps. However it allows analysis of binding parameters independently from enzymatic activity. To date relatively cumbersome cell culture, *ex-vivo* methods and the mouse bioassay can evaluate all three functions necessary for toxicity (binding, translocation, endoprotease activity).

In this study, we used a SPR biosensor to establish a simplified automated assay for BoNT/B binding to exoSYT2. Using two different BoNT/B sources K_D_ values derived from kinetic constants were close to those using ^125^I-BoNT/B at equilibrium with nerve terminals or SYT2/GT1b proteoliposomes, thus validating the accuracy of our approach^14, 15^. Interestingly, the exosomal approach circumvents time-consuming detergent reconstitution procedures required to prepare proteoliposomes. Even though exosome purification involves multiple steps, a single exoSYT2 preparation provides sufficient material for more than 100 binding tests and can be frozen for long periods without degradation. Curiously, HcB, the BoNT/B domain that binds to SYT2, displayed a higher affinity than BoNT/B holotoxin, owing to an extremely slow dissociation phase. The difference in off-rate may reflect a reduced spatial restriction for HcB compared to BoNT/B, to accommodate the double SYT2/GT1b receptor^26^. Alternatively this might be explained by differences in affinity of BoNT subtypes, as it was reported that BoNT/B2 may be more active than BoNT/B1^29^. This finding could be of specific importance since HcB has potential for the development of protective recombinant vaccines against botulism, and to target specific drugs to nerve terminals^35, 36^.

In conclusion, we have developed a new approach to express membrane proteins in exosomes providing detailed information on ligand-receptor interactions using biosensors. We focused on measuring the affinity of BoNT/B for its receptor, as precise determination of binding parameters can constitute a key factor in using BoNTs as therapeutic agents. Exosomes thus constitute a useful nanoscale system for manipulating membrane proteins in robust soluble structures for analysis of molecular interactions, vaccine production as well as screen for drugs that specifically target receptors embedded in exosomal membranes.

## Methods

### Chemical and reagents

Unless otherwise stated, chemicals and Nunc Maxisorp 96 well ELISA plates were from Sigma. BoNT/A (A1 Hall strain; 1 mg/ml) and BoNT/B (B1 Okra strain; 1 mg/ml) neurotoxins with non-toxic accessory proteins, subsequently designated as BoNT, were obtained from Metabiologics (Madison, WI). Pure holotoxins type A (A1; 0.9 mg/ml), B (B1; 1 mg/ml) and E1 (0.7 mg/ml) were generously provided by S. Kozaki^37^. All experiments were performed in accordance with French and European guidelines for handling botulinum neurotoxins. His-tagged HcB (from BoNT/B2 heavy chain, amino acids 861–1291)^29^, HcA (amino acids 1096–1296)^38^ as well as affinity-purified polyclonal anti-BoNT/B antibody^16^ were described previously. Polyclonal anti-BoNT/A and anti-BoNT/B antibodies were purchased from Metabiologics. The following monoclonal antibodies were from the following sources: CD9 (MEM-61, Abcam), CD63 (TS63, Abcam), CD81 (1D6, Abcam), GT1b (MAB5608, Millipore), GD1a (A2507, AMSbio), Kv1.2 (Cl. K14/16, NeuroMab), CXCR4 (44717, R&D system), AMA1 (clone CL22 gift from J. F Dubremetz). Polyclonal antibody anti-SYT2, mAb 8G2b and mAb5b7a directed against a 20 amino acid peptide corresponding to the N-terminal rat SYT2 sequence and mAb 1D12 recognizing a cytosolic domain of SYT1/2 were generously provided by M. Takahashi^12, 39^. Goat anti-GST polyclonal antibodies were from GE Healthcare Life Sciences and HRP coupled secondary antibodies were from Interchim. TMB (3,3′,5,5-tetramethylbenzidine) was from Uptima. ^125^I α-dendrotoxin was from PerkinElmer, gangliosides GT1b from Calbiochem. Recombinant SDF-1α protein and AMD3100 were obtained from R&D systems. Healthy human sera were obtained from the “Etablissement Français du Sang”. HEK cells were from ATCC: [HEK 293T/17] (ATCC^®^ CRL-11268^™^).

### DNA

Nucleic acid sequences encoding human synaptotagmin 1 (SYT1h; NP_005630.1), rat synaptotagmin 2 (SYT2r; NP_036797.1), Toxoplasma gondii apical membrane antigen 1 homolog (AMA1Tg, GenBank: AF010264.1), human C-X-C motif chemokine receptor 4 (CXCR4, GenBank: AY242129.1), and human Kv1.2 channel (XP_011539702.1) were cloned in the expression plasmid pCA-IZ (Ciloa Co.). These coding sequences were inserted 5′ of and in frame with the DCTM coding peptide (5.3 kDa) sequence both being upstream of an IRES that allows the translation of a Zeocin resistance gene. Control exosomes (exocontrol) were produced and purified from cells transfected with pCA-IZ without additional insert. CXCR4exosomes were produced also from pCILA-IZ plasmid devoid of DCTM encoding sequence.

### Transient transfection and generation of stable cell lines

Plasmids were transfected in HEK 293T cells (about 1.10^6^ per well) using JETPrime® (Polyplus Transfection). In order to generate stable cell lines for SYT1h and SYT2r, transfected cells were plated at very low density, cultured for 10–14 days in complete culture medium (DMEM (GIBCO 41965-039) supplemented with 2 mM Glutamax (GIBCO 35050-061), 20 µg/ml Gentamicin (GIBCO 17510-080) and 10% heat-inactivated fetal bovine serum in the presence of Zeocin (400 µg/ml). Clonal cell populations were isolated using an agarose-based cloning rings anchoring method^40^.

### Exosome production and purification

Exosomes were produced and purified either from transiently or stably transfected cells (60–250 × 10^6^). The culture medium was collected after 2 to 3 days and exosomes enriched as follows: Supernatants of two consecutive centrifugations 10 min at 300 g and 15 min at 10.000 g were filtered through 0,22 µm membrane filters (Millipore Stericup filter) and centrifuged for an additional 2 h at 220.000 g using Beckman LB-70M ultracentrifuge. Pellets were resuspended overnight in 2 ml EB (137 mM NaCl, 2.7 mM KCl, 10 mM Na_2_HPO_4_, 1.8 mM KH_2_PO_4_ pH 7.4) and centrifuged again. The final pellet was resuspended in 1 ml EB. For large production (>400 × 10^6^ cells) of exosomes from synaptotagmin expressing stable cell lines, ultrafiltration (300 kDa MWCO), centrifugation and gel filtration were performed to concentrate and increase exosomes purity^41^. Similar production method was performed with mock-transfected HEK cells to prepare control exosomes (exocontrol). Exosomes were aliquoted and stored at −20 °C.

### Exosome sizing

0.1 μg of exosomal solution diluted in Milli-Q® ultrapure water was sized using Nanoparticle Tracking Analysis (NanoSight NS300). Data were collected for 60 s and analyzed using NanoSight NTA 2.3 software.

### Western blots

3 µg of each exosomal preparation were analyzed by Western blotting, revealed with the ECL system (Amersham Biosciences). Peptide -N-Glycosidase F (PNGase F) deglycosylation of SYT1 and SYT2 exosomal preparations was carried out following manufacturer’s instructions (New England Biolabs).

### ^125^I-dendrotoxin binding assays

2 µg Kv1.2-expressing exosomes (exoKv1.2) were incubated at room temperature for 60 min with 0.7 nM ^125^I-dendrotoxin in 100 µl 0.1% TBSA (10 mM Tris-Cl pH 7.4, 140 mM NaCl,  0.1% bovine serum albumin (BSA)) in the absence or presence of 1 µM unlabelled dendrotoxin. Incubation was followed by rapid filtration over glass-fiber filters (Whatman GF/B) pretreated with 0.3% aqueous polyethyleneimine. Filters were washed 3 times with 0.1% TBSA at 4 °C and counted on a gamma counter.

### ELISA detection of exosomal markers

ExoSYT2 (2 µg/well) in 0.1% TBSA were captured on 3% TBSA blocked ELISA wells, precoated with polyclonal anti-SYT2 or control anti-GST antibodies. Steps were preceded by washing with 0.1% TBSA. The following mAb antibodies were added and incubated for 1 h at 37 °C (2 µg/ml): anti-CD9, anti-CD63, anti-CD81, anti-synaptotagmin2 (8G2b), or anti-synaptotagmin 1/2 (1D12) in 0.1% TBSA. Donkey anti-mouse-HRP conjugated antibodies were then used and HRP activity measured at OD_450nm_ after addition of TMB and stopping the reaction by acidification.

### ELISA detection of BoNT/A and B binding to exosomes

Exosomes (1 µg protein per well) were directly adsorbed in ELISA wells in 200 mM Na_2_HCO_3_ pH 8.9. Steps were preceded by washing at room temperature using 0.1% TBSA. Wells were blocked with 3% TBSA and GT1b (4.5 µM) was added in TBS and incubated for 30 min at 37 °C. Plates were then incubated for 1 h at 37 °C with BoNT/B or BoNT/A in complex (10 nM) or HcB. Depending on experiments, BoNTs were added in 0.1% TBSA with or without 25% healthy human serum. Polyclonal anti-BoNT/B antibodies (1 µg/ml) were added and incubated for 1 h at 37 °C. Goat anti-rabbit-HRP conjugated antibodies were added and HRP activity measured at OD_450nm_ after addition of TMB and stopping the reaction by acidification.

### ELISA detection of CXCR4

CXCR4 (ExoCXCR4) or DCTM-fused CXCR4 expressing exosomes (exoCXCR4/DCTM) were produced and purified under the same conditions. Both exoCXCR4 and exoCXCR4/DCTM (37,5 ng/well) were directly adsorbed in ELISA wells. Wells were blocked using Phosphate buffered Saline (PBS) containing 3% BSA, for 1 hour at 37 °C. Every subsequent step was preceded by washing using PBS supplemented with 0.05% Tween. Anti- CXCR4 (1 µg/ml) antibodies were incubated for 2 h at 37 °C in PBS and secondary HRP conjugated antibodies were added. HRP activity was measured at OD_450nm_ after addition of TMB and stopping the reaction by acidification.

### ELISA detection of gangliosides

ExoSYT2 were captured and gangliosides detected using mAb anti GT1b (ascites diluted at 1/4000) and GD1a (2.5 µg/ml). Non-specific binding measured from control wells without exosomes was subtracted. Positive control for GT1b was performed by pre-incubating exosomes with exogenous GT1b 8 µM. HRP activity on secondary antibodies was measured at OD_450nm_ after addition of TMB and stopping the reaction by acidification.

### Surface Plasmon Resonance (SPR) experiments

SPR measurements were performed at 25 °C using Biacore 3000 or Biacore T200 (GE Healthcare) apparatus. HBS (10 mM 4-(2-hydroxyethyl)-1-piperazine ethane sulfonic acid (HEPES)/NaOH pH 7.4, 150 mM NaCl) was used as running buffer and sensorchips were either CM5 from GE Healthcare or chip without dextran from Xantec (2D carboxymethyldextran surface CMDP). Sensorchips were functionalized by covalently coupling mAb antibodies recognizing CD81, CXCR4 or SYT2 (5B7a) using standard amine coupling chemistry (800–1200 RU on CMDP chip). Exocontrol or exoAMA1 were captured on flow cells functionalized with anti-exosomal marker CD81 antibody and used as negative control. ExoSYT2 were captured using 5B7a or anti-CD81 antibodies. Exosomes (0.3 mg/ml) were injected at a flow rate of 1 µl/min. The amount of bound exosomes varied between experiments and ranged from 500 to 2500 RU. For experiments involving exoSYT2, GT1b (1 µM) was added to pre-immobilized exosomes with a flow rate of 10 µl/min and the loading stopped upon reaching 1/3 of the exosome mass. Taking a mean molecular weight of 50 kDa for proteins and 2.2 kDa for GT1b, a protein to lipid ratio of 2 (w:w) similar to synaptic vesicles^42^, we calculated a molar ratio GT1b/total proteins of 10. Exosomes/GT1b were used for binding experiments at least 2 hours after GT1b loading. Five different concentrations of analytes, prepared by twofold dilutions ranging from 1 to 16 nM or 2 to 32 nM, were placed on the Biacore sample rack at 4 °C and assayed across the control and SYT2 exosomes surfaces at a flow rate of 30 µl/min. The data obtained from the control flow cell were automatically subtracted from experimental measurements to yield the specific signal. Data were analyzed using Biacore T200 Evaluation software 2.0 or BIAevaluation software 4.1 (GE Heathlcare). The single-cycle kinetic method was used in affinity measurements and K_D_ values (k_off_/k_on_) were calculated with the 1:1 titration kinetic binding model. Chips were regenerated using 100 mM octylglucoside as described^43^ and used several times.

### Statistical analysis

Values presented are mean ± standard error of the mean (SEM) or mean ± standard deviation (SD). Statistical differences were assessed using the Wilcoxon matched-pair test. A p value < 0.05 was considered to be significant.

## Electronic supplementary material

Supplementary Information
