# Supplementary material for: Affinity biosensors using recombinant native membrane proteins displayed on exosomes: application to botulinum neurotoxin B receptor
Source: Sci Rep. 2017 Apr 21;7:1032. doi: 10.1038/s41598-017-01198-1 (PMC5430821; doi:10.1038/s41598-017-01198-1)
Supplement: Supplementary file 1 — Supplementary Information [file 41598_2017_1198_MOESM1_ESM.pdf]

Supplementary methods

Affinity biosensors using recombinant native membrane proteins displayed on exosomes: application to botulinum neurotoxin B receptor

Richard Desplantes[a,c], Christian Lévêque[a,c], Benjamin Muller[d], Manuela Lotierzo[d], Géraldine Ferracci[b,c], Michel Popoff[e] , Michael Seagar[a,c], Robert Mamoun[d], and Oussama El Far [a,c*]

Enrichment of recombinant molecules in exoSYT2 and exoCXCR4:

CXCR4 or SYT2/GT1b exosomes were captured using anti-CXCR4 or anti-SYT2 antibodies immobilized on a chip without dextran. Rmax in RU units was then measured by injecting anti-CXCR4 antibodies or anti-SYT2 antibodies (5B7a) at a saturating concentration (330 nM). Previous studies have shown that anti-VAMP mAb injected at saturation over synaptic vesicles immobilized on a sensorchip give a value of 0.084 RU of mAb per RU of immobilized synaptic vesicle (Ferracci et al. 2004). The number of copies of VAMP per synaptic vesicle was then estimated to be 70 (Takamori et al. 2006). Taking into account that exosomes have a diameter twice that of synaptic vesicles, for the same mass of vesicles immobilized on a chip, there will be 4-fold less exosomes. As anti-SYT antibody binding gave 0.07RU per RU of exosomes, one can calculate that exosomes express at least 3.3 times (4 x 0.07/0.084) more copies of SYT than VAMP in synaptic vesicles, i.e 231 copies.

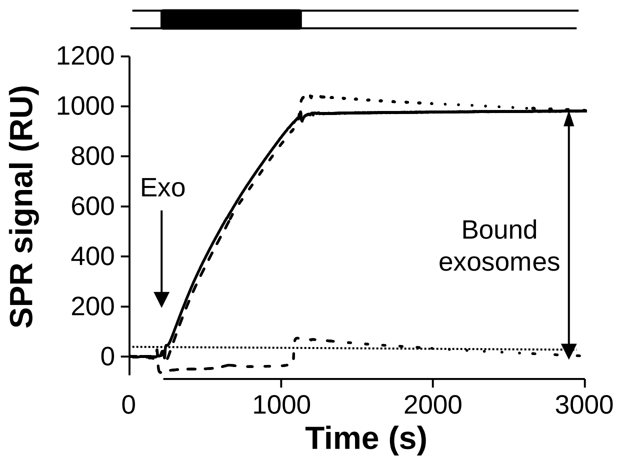

Fig. S1. Specific immobilization of exoSYT2 on a sensor chip. Exosomes were injected (black bar) over immobilized anti-SYT2 antibodies 8G2b (upper dashed trace) and control IgG (lower dashed trace). The specific binding (continuous line) corresponds to the subtraction of the lower from the upper dashed trace. Exosomes remained stably immobilized during running buffer injection (open bar).

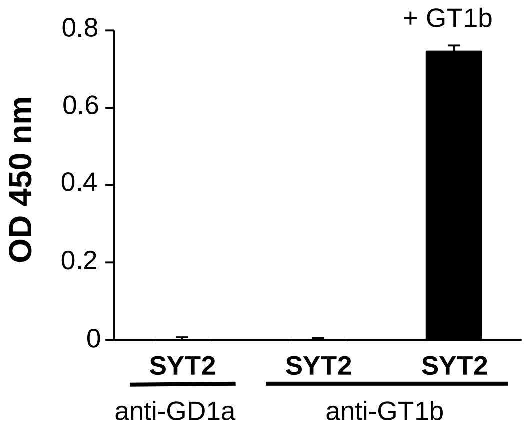

Fig. S2. exoSYT2 do not express GT1b or GD1a**.** ExoSYT2 were captured on ELISA plates using polyclonal anti-SYT2 antibodies. Anti-GT1b and GD1a antibodies were used to detect endogenous gangliosides. Neither GT1b nor GD1a were expressed while GT1b was detected when added exogenously. Representative of 2 independent experiments.

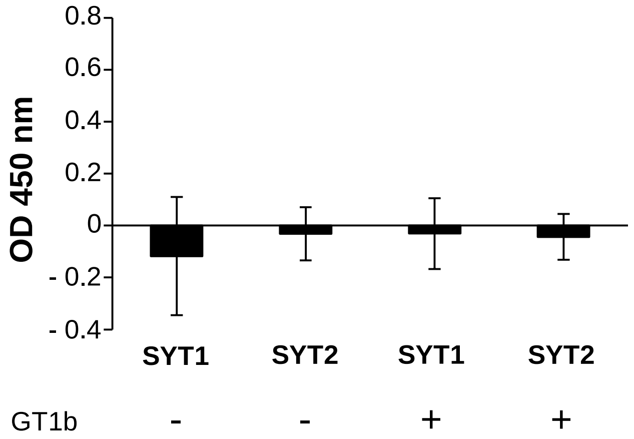

Fig. S3. Absence of BoNT/A binding to exoSYT1 and exoSYT2. BoNT/A (10 nM) was incubated with SYT1 and SYT2 exosomes in the presence or absence of GT1b and binding detected using anti-BoNT/A antibodies. Non-specific binding to control exosomes has been subtracted. Representative of 2 independent experiments.
